# Evaluation of the Mozambique Antiretroviral Therapy (MozART) database as an antiretroviral therapy patient surveillance system, 2017-2018

**DOI:** 10.11604/pamj.2022.42.137.28931

**Published:** 2022-06-21

**Authors:** Neusa Vanessa Fernandes Abdul Fataha, Sandra Gaveta, José Carlos Langa, Auria Ribeiro Banze, Jahit Sacarlal, Erika Valeska Rossetto, Cynthia Semá Baltazar, Timothy Allen Kellogg

**Affiliations:** 1 Mozambique Field Epidemiology and Laboratory Training Program, Maputo, Mozambique,; 2 National Institute of Health, Maputo, Mozambique,; 3 Faculty of Medicine, University of Eduardo Mondlane, Maputo, Mozambique,; 4 MassGenics, Assigned to Centers for Disease Control and Prevention, Maputo, Mozambique,; 5 University of California San Francisco (UCSF), Institute for Global Health Sciences, San Francisco, California, USA

**Keywords:** Health information systems, evaluation, HIV, antiretroviral therapy, Mozambique

## Abstract

**Introduction:**

Mozambique antiretroviral therapy is a database used to monitor patients receiving antiretroviral treatment (ART). This study's objective was to evaluate the system for the purpose to monitor patients receiving ART.

**Methods:**

data from 287,052 patients who started ART from January to December 2017 were verified, and retention in care was assessed for 2018 in Mozambique. The Centers for Disease Control and Prevention guidelines for evaluating public health surveillance systems were used to conduct the evaluation. Simplicity, flexibility, data quality, representativeness and stability attributes were evaluated.

**Results:**

a total of 93% (266,880/287,052) of patients on ART were adults ≥15 years old, and 65% (186,677/287,052) were female. The system was complex, it involved four organisations and its management was online. Data quality was moderate with 19% (1,533,885/8,037,456) of empty variable fields, 0.04% (123/287,052) observations with birth date later than the initial ART date, 0.2% (424/287,052) and 23% (68,039/287,052) with initial ART date and diagnosis date, later than the next ART pickup date. Nationally, 19%(31/161) of the districts did not have data in the information system. MozART cover health facilities with electronic patient tracking systems. Hence did not represent all patients on ART. While it was not possible to add variables of the electronic patient tracking, the system was stable as neither data or server interruptions were reported.

**Conclusion:**

the system was useful, stable, with moderate data quality, complex, not flexible and not representative. We recommend to health facilities and partners to develop and distribute procedures for data validation and completeness and report all patient tracking variables in the system.

## Introduction

Human Immunodeficiency Virus (HIV) has become a major epidemic in Mozambique and remains a global concern [1]. As of 2019, 38 million people were living with HIV (PLHIV) worldwide, and among PLHIV, 67% were on [2]. Africa is the most affected continent globally, with 25.6 million of PLHIV, and over 440,000 deaths related to acquired immunodeficiency syndrome in 2019 [3]. In Mozambique, approximately 2.2 million people are infected with HIV [4]. Based on the last population survey in 2015, the HIV prevalence of PLHIVamong adults was 13.2% [5]. In Mozambique, the prevalence of adults, men and women with 15-49 years was 12.4% [6]. In recent years, the provision of care has become an acute problem due to the increased need for antiretroviral treatment (ART). In 2016, Mozambique adopted recommendations from the World Health Organization (WHO) to start a universal ART to all PLHIV regardless of clinical status [7]. Long term, sustained treatment can significantly reduce replication of HIV among infected persons, thereby reducing the number of new infections [8]. Follow-up of patients on ART is essential to measure and assess retention in care and treatment [9]. Retention is an important metric that measures patient engagement to care and treatment at specific time points. Retention can be measured at 6, 12, 18, 24 months or longer time intervals [10]. In 2019, about 67% of patients who started ART in Mozambique remained active after 12 months [4]. Due to the loss of patients on ART over time, strategies are needed to retain patients in the health facilities and identify reasons for loss to follow up [10]. The Mozambique Antiretroviral Therapy (MozART) database is a national patient-level ART information system that records and stores data for research, monitoring and surveillance purposes. MozART started operating in 2005 following the migration of paper format records to the electronic patient tracking systems (EPTS). Patient-level data is entered retrospectively into the Open Medical Record System (OpenMRS) in health facilities. The Open MRS is a medical record storage system that records patient tracking information in an electronic database management system. The purpose of this evaluation was to determine whether MozART can monitor ART retention outcomes efficiently and effectively. The results of this assessment are intended to contribute to improving the functioning of the system.

## Methods

**Overview of the MozART database, 2017-2018:** MozART is an information system that regularly records patient information such as clinical, laboratory and ART information and can be important for monitoring whether patients comply with their ART pickup dates. MozART's operation began in 2005, when partners abandoned the physical paper format for the EPTS at the health facilities. The selection criteria to install the EPTS at the health facilities is a minimum of 500 patients on ART, but not all healh facilities have EPTS despite having 500 patients on ART. The MozART, an ART patient information system, stores data for patient tracking, analysis, research interpretation and surveillance.

**Source of data collection:** secondary data from MozART with information from patients on ART were used for the evaluation. The evaluation was conducted with a database of 28 variables.

**Delineation of the MozART assessment:** all HIV-positive patients registered within MozART who started ART between January and December 2017 were included in the study population. Retention in care and treatment was assessed at 12 months after ART initiation. This evaluation followed the Centers for Disease Control and Prevention (CDC) updated 2001 guidelines for evaluating public health surveillance systems [11]. The evaluation focused on evaluating MozART based on the following attributes: usefulness, simplicity, flexibility, data quality, representativeness and stability (Table 1). The usefulness attribute was evaluated based on actions taken due to the analyses and interpretation of the data. Our evaluation of the simplicity of MozART focused on the number of data transfer levels, data dissemination from the health facilities and method of data handling. Flexibility was evaluated by assessing how the system handled the introduction of new variables. The EPTS follow-up form and the MozART variables were reviewed to check the differences between MozART and EPTS variables. Data completeness was calculated by the total number of fields filled divided by the total number of fields available in MozART. The fields were considered filled when they presented data. Data quality was evaluated by comparing date variables (the date of diagnosis, date of the next ART pick-up, date of birth and date of ART initiation), the percentage of men recorded as pregnant, and the number and percentage of duplicate numeric identification numbers (NID). The representativeness of MozART was evaluated using the distribution of patients by gender and age group and reviewing the number of patients in ART per month, by province, and by district, comparing this distribution to the official ministry of public health figures. The system's stability was evaluated based on the number of times the system had been unavailable in a month in 2018, and MozART changes from CDC/United States Agency for International Development (USAID) to National Health Institute.

**Table 1:** criteria and parameters for evaluating attributes of MozART, January 2017-December 2018

Attributes	Criteria to be analyzed	Parameter	Scoring	Score
Simplicity	Number of data transfer levels	3 levels - simple = 0 ≥ 4 levels - complex = 1	0 to 3 points Simple: ≤ 1 point complex: > 2 point	4 levels - complex = 1
	Data dissemination	Ms access = 0 MySQL to Ms access - complex = 1		Different data - complex = 1
	Data handling method	Offline - simple = 0 online -complex = 1		offline - simple = 1
Flexibilility	Inclusion of new variables (control of medical prescriptions and ART side effects)	flexible = 1 not flexible = 0	flexible = 1 not flexible = 0	not flexible = 0
Data quality	Proportion of missing data	< 10% - excellent = 2 10 - 70% - moderate = 1 >70% - poor = 0	0 to 12 point total ≥ 11 high 5 - 10 moderate ≤ 4 low	19% - moderate = 1
	Diagnostic date later than the next ART pick up date.	< 10% - excellent = 2 10 - 30% - moderate = 1 ≥ 30% bad = 0		23% - moderate = 1
	Birth date later than the date of initiation ART			0.04% - excellent = 2
	ART initiation date later than the next ART pick up			0.15% - excellent = 2
	Men registered as pregnant			2% - excellent = 2
	Duplicate NID			1% - excellent = 2
Representativity	Person: number of patients by gender and age group	Representative = 1 not representatie = 0	0 - 4 points: 0 - 4 = not representative >5 = representative	Representative = 1
	Time: number of patients in ART by month in 2017.			Representative = 1
	Place: number of patients in ART by province.			Representative = 1
	Place: number of patients in ART by district.			Not representative = 0
	Comparing retention rate and annual report on HIV/AIDS-related activities, 2018			Representative = 1
Stability	Number of times that system has been unavailable	Classification: high = 0 Low ≥ 1	Scoring 0 - 2	High = 0
	Changes in MozART from CDC/USAID to NHI			High = 0

**Ethical approval and consent to participate:** this was an evaluation of a surveillance system, it did not constitute research and therefore no ethical consent was required. This evaluation did not involve direct human subjects, consent was not required from individual patients.

## Results

**Figure 1:**
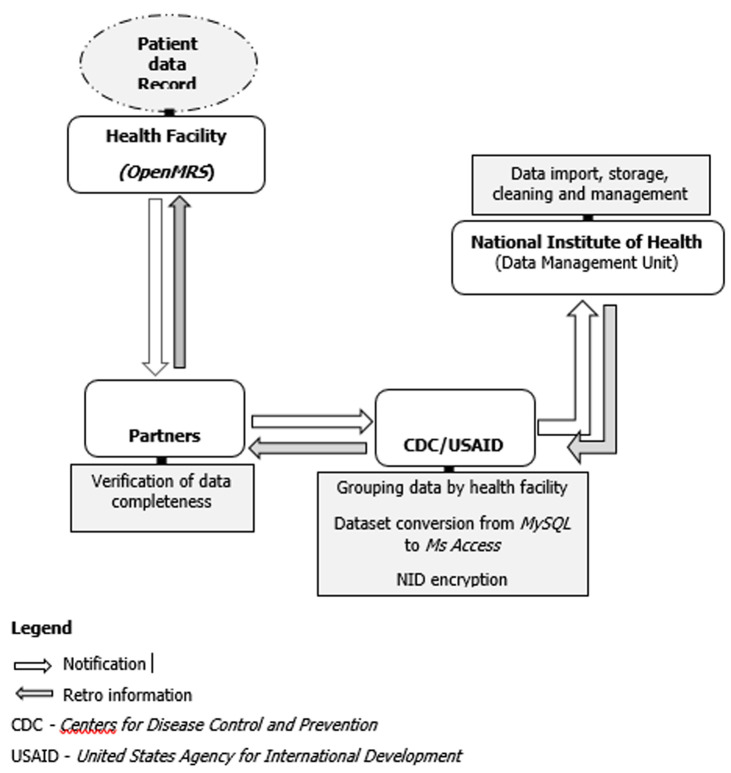
MozART information flow diagram, Mozambique - 2017- 2018

**Overview of the system:** the database used for evaluation had 28 variables and 287,052 observations of patients who initiated ART in the 2017. Patient data were entered into the Open MRS implemented at the health facilities. This system tracked patient events such as new and follow-up visits, and diagnoses as they occur. Open MRS data were stored in a MySQL database management system. Clinical partner organizations providing HIV services in Mozambique's provinces and sent electronic data to CDC and USAID. At the time of this evaluation, partners were sending data quarterly, but during the COVID-19 pandemic data submissions are now submitted every 6-months. The partner was responsible for validating and grouping data by health facilities, converting EPTS data (MySQL) to MozART dataset (MS Access) and NID encrypting. Once data were sent, CDC and USAID compared it with the master health facilities list to ensure patient data reporting from all facilities at every reporting period. Every quarter the data were transferred to the Data Management Unit at National Institute of Health for storage, cleaning, and future management. While partners and health facilities did not receive the MozART data, they received feedback about transmission challenges (Figure 1). As of April 2019, the MozART database contained 737 health facilities, 26 data tables, 86 variables and 1,952,757 observations captured for each patient visit. MozART's coverage in 2018 was 51%.

**Usefulness:** MozART data were used to conduct data analyses and produce reports and bulletins, such as the retention and viral suppression bulletin that the National Institute of Health published. Data analysis and interpretation using MozART data, HIV intervention program planning, performance monitoring, and improved decision making in the HIV program have been carried out.

**Simplicity:** four institutions were involved in the functioning of MozART, the national health facilities, CDC, USAID and National Institute of Health. The data originated from health facilities responsible for Open, MRS. The data transfer took place when partners managed the Open MRS data transformed from MySQL to Ms Access. The CDC/ SAID and National Institute of Health managed MozART data. Data handling and information, data cleaning procedures, and data sets for analysis were conducted online. As a result, the MozART was considered complex, the final score was three.

**Flexibility:** although it was possible to find variables in the EPTS system, such as control of medical prescriptions, with the information that the medicines were not available if the prescription expires and clinical manifestations of side effects which were categorized as non-serious, serious and life-threatening it was not possible to add these variables to MozART. The information was registered in EPTS but not in MozART, as a result, the system was considered not flexible.

**Data quality:** about 287,052 observations of patients were enrolled in ART during the MozART evaluation period. The system included 28 variables and 8,037,456 fields in a total of which 19% (1,533,885/8,037,456) fields were missing data, and thus the quality of the data in the information system was moderate (Table 1). Of the observations 0.04% (123/287,052) had a recorded birth date later than their initial ART date, 0.15% (424/287,052) had an initial ART date recorded as later than the next ART pick-update and 23% (68,039/287,052) had the diagnosis date after the next ART pick-up date. We found that 2% (5,155/287,052) of men were recorded as pregnant. The information system did not prevent the completion of the pregnancy variable for male patients. The MozART system identified patients by a unique NID. To properly follow patients over time, each patient needs to be uniquely identified with the same NID to merge future clinic visits into the baseline clinical. In this analysis, duplicate NIDs were patients with the same identification number. Duplicated NIDs were further evaluated by comparing other demographic information such as age, gender, and clinical data. Out of the 287,052 observations, 1% (4,197/287,052) had a repeating NID indicating two or more patients with the same identity number. Additionally, a random sample of 2.3% (100/4197) with repeating NIDs was assessed by line-listing age, sex, and clinic information. About 65%(65/100) of NIDs had the same analysed information suggesting they may be the same person, while 29% (29/100) had different information suggesting that they may be different people assigned the same NID. The remaining 6% (6/100) could not be determined as one or more of the three variables were missing. The information system had moderate data quality.

**Figure 2:**
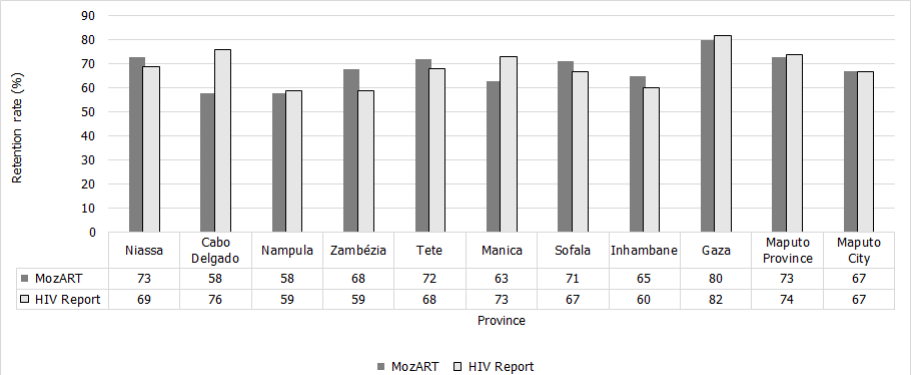
number of patients retained at 12-months in MozART compared to Ministry of Health data in Mozambique, 2018 (N = 181.795)

**Representativeness:** the MozART was representative in all age groups, 93%; (266,880/287,052) of patients on ART were adults ≥15 years old, and 65% (186,677/287,052) were female. In 2017, the distribution of patients on ART was similar across all months. Zambézia province had 22% (64,042/287,052) number of patients on ART. Nationally, 19% (31/161) of the districts did not have data in the information system. Districts with EPTS also had health facilities without the system. Of health facilities at the national level, 47% (688/1,455) were included in MozART. In the country, 328,071 patients started ART in 2017 [12]. MozART included data for 287,052 patients who started ART in 2017, thus 13% (41,019/328,071) of patients who started ART in that year were not included in MozART and 87% (287,052 /328,071) were included. We compared retention data from the information system and the Ministry of Health Annual Report on HIV/AIDS-related activities, 2018 [12] and found small differences in the percentage of patients retained in ART by province (Figure 2). The MozART was not representative, the final score was four.

**Stability:** MozART data was officially transferred from CDC to National Health Index (NHI) on 13^th^ July 2017, period over which the system data were evaluated. A total of 287,052 patients started ART in 2017, of which 55% (157,999/287,052) enrolled in ART before the transfer, and 45% (129,053/287,052) enrolled after. The CDC and NHI continued to manage data, hence data transfer did not change data management and analysis processes. Data interruption, unscheduled system and server interruptions were not reported in 2017-2018. We, therefore, characterized MozART as a stable system.

## Discussion

Information systems for PLHIV can be significant sources of public health data, as they can facilitate the monitoring of patients on ART and surveillance of clinical events related to HIV infection [13]. The attributes evaluated were independent; however, the improvement of one could interfere with the other's performance [14]. Several institutions were involved in the data transfer, information on ART patients was available in MozART as required for monitoring purposes. However, because sending data required several institutions, the system was considered complex and subject to data quality problems. The evaluation of the data makes it possible to identify areas with inadequate ART coverage, which demonstrated the system's importance for planning, actions and allocating human, financial, and material resources. The absence of EPTS in some health facilities resulted in inadequate ART coverage in MozART. The selection criteria for EPTS installation at health facilities was at least 500 patients on ART, but not all health facilities had EPTS although they had 500 patients on ART. The transfer procedure for converting EPTS (MySQL) data to the MozART (MS Access) data set probably had a substantial effect on the quality of data. Due to the unavailability of the documentation of the MozART transfer code, the amount and the type of data flow from EPTS to MozART was not known. The small differences in the percentage of patients retained in ART by province and HIV report suggested that patient-level MozART estimates were in agreement with program data, which can be due to the period over which retention was assessed. Some variables presented inconsistency data and variable fields not filled in MozART. Hence, it may be developed and distributed data validation procedures and completeness at health facilities.

## Conclusion

The information system met its objectives as it monitored patients on ART. The system was complex, was not flexible, the data quality was moderate, was not representative and it was stable. We recommend to health facilities and partners to develop and distribute procedures for data validation and completeness and report EPTS variables in MozART. Regular monitoring of retention would be useful for evaluating the effectiveness of ART treatment programs in Mozambique.

### What is known about this topic

- *The patient surveillance system on ART shows that it regularly records information on ART and monitors whether patients comply with antiretroviral with drawal dates;*
- *MozART analyzes pre-tarv and post-tarv outcomes for adults and children retrospectively with the goal of identifying groups of patients at risk of ART dropout;*
- *Clinical data collected through EPTS have the potential to provide national estimates of patient outcomes, including retention*.

### What this study adds

- *Need to add missing variables to the database that are in the EPTS in order to collect more characteristics of patients on ART;*
- *This evaluation shows that although MozART has potential for providing routine surveillance for ART outcomes, issues as data quality and representativeness may limit the usefulness of the surveillance system at the national level*.
